# Revising a laparoscopic appendicectomy set to reduce reliance on disposable surgical instruments: supporting the transition to sustainable surgical practice

**DOI:** 10.1308/rcsann.2023.0015

**Published:** 2023-04-13

**Authors:** PL Labib, BSM Ford, M Winfield, WJ Douie, A Kanwar, G Sanders

**Affiliations:** University Hospitals Plymouth NHS Trust, UK

**Keywords:** Appendectomy, Health care economics and organisations, Laparoscopy, General surgery, Surgical procedures, Operative

## Abstract

**Introduction:**

After excluding anaesthetic gases, around one-third of carbon emissions from surgical procedures are from consumables. This sustainable quality improvement project revised the laparoscopic appendicectomy surgical set at a large teaching hospital, with the aim of reducing unnecessary usage of disposable laparoscopic ports and surgical instruments.

**Methods:**

A prospective audit of 25 consecutive laparoscopic appendicectomies (5% of annual appendicectomies performed at the Trust) was conducted to assess use of disposable instruments. The financial and environmental costs of the five most commonly used disposable instruments were calculated and annual cost of current practice determined. A revised surgical set was created to include additional reusable instruments and new reusable ports. A reaudit of disposable surgical instrument usage was conducted and the financial and environmental impact of the new set compared with the results from the initial audit.

**Results:**

A total of 109 disposable instruments were opened in 25 appendicectomies, costing an estimated £49,656 and 692kg CO_2_ equivalent (CO_2_e) annually. Following rollout of the revised appendicectomy set, there was a significant reduction in disposable instrument usage (median four versus one instruments per case, *p*<0.00001). The revised set is predicted to reduce annual disposable instrument usage from 2,180 to 705 instruments (68% reduction), saving £219,452 and 3.02 tonnes CO_2_e over the estimated seven-year lifecycle of the reusable instruments.

**Conclusions:**

Updating a laparoscopic appendicectomy set to include additional/new reusable instruments can lead to a marked reduction in disposable surgical instrument usage. This results in significant projected financial and CO_2_e savings.

## Introduction

**Figure 1:**
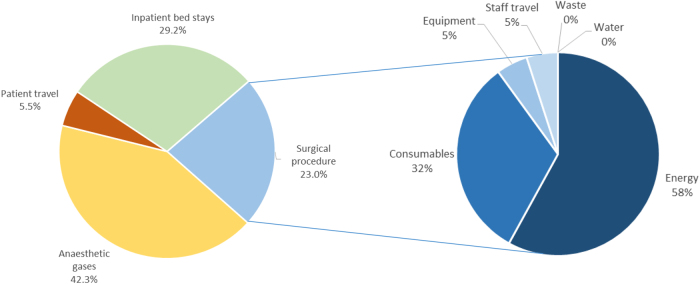
Estimated sources of carbon emissions from surgery in England^2^

In 2022, the NHS legally committed to achieve net zero carbon emissions by 2045.^1^ It is estimated that 77% of emissions from surgery are from anaesthetic gases, inpatient bed stays and patient travel, and 23% from the surgical procedure itself (Figure 1).^2^ From the procedure, 58% of emissions are attributable to energy consumption (primarily theatre ventilation systems) and one-third to consumables. Whereas there has been a national focus on reducing emissions from many of these sources (e.g., reducing use of volatile anaesthetic gases, virtual outpatient clinics and day case surgery), a coordinated drive to reduce surgical consumable reliance has been lacking. Reasons for this include no reusable alternatives (e.g., laparoscopic gas tubing), surgeon preference for particular equipment and the habitual nature of many practical elements of surgical practice (‘It’s what we’ve always done’). In addition, the capital investment required for the purchase of reusable instruments can be perceived as an expenditure rather than a potential net cost saver by reducing consumable reliance.

This sustainable quality improvement (susQI) project aimed to assess the environmental and financial impact of updating the laparoscopic appendicectomy set in a large teaching hospital, with a view to reducing reliance on single-use surgical instruments and ports.

## Methods

### Surveys of current surgical practice

An online survey (Google Forms) asked colleagues about their current laparoscopic port and disposable instrument usage. A second survey asked colleagues their reasons for using single-use instruments/ports during laparoscopic appendicectomies, and suggestions regarding which rarely used instruments could be removed from the set.

### Audit

A prospective audit of 25 consecutive appendicectomies (5% of annual appendicectomies performed at our Trust) was conducted to assess single-use instrument usage during laparoscopic appendicectomies. To avoid recall bias, audit forms were completed by theatre staff rather than surgeons. Consumables with no reusable alternative (e.g., laparoscopic gas tubing) were excluded.

### Assessment of financial and environmental cost of current practice

The procurement team provided unit prices for the five most commonly used disposable instruments; 5mm ports (Kii Fios, Applied Medical), 12mm ports (Kii Fios, Applied Medical), Johann graspers (Espiner disposable lap intestine forceps, Fannin Ltd), Maryland bipolar forceps (bipolar Maryland dissector, LocaMed Ltd) and laparoscopic scissors (disposable curved Metzenbaum scissors, CJ Medical). Based on the audit results, an estimated annual financial cost from using these instruments during appendicectomies was calculated.

A cradle-to-grave carbon footprint analysis was conducted on the above five disposable instruments that followed the attributional approach of the Greenhouse Gas Protocol Product Lifecycle Accounting Reporting Standard. This took into account the CO_2_ equivalent (CO_2_e) emissions associated with the extraction of raw materials, manufacture, transport to hospital and disposal of each disposable item and its packaging. The composite materials used in each disposable item were determined from the product packaging, emailing companies directly and academic publications. Emissions factors for each material and transport method were taken from the UK government greenhouse gas emissions factors 2021 report and the Inventory of Carbon and Energy database.^3,4^ The copper wire emission factor was calculated based on a life cycle analysis from the European Copper Association.^5^ Overseas production was presumed to arrive from factory to nearest port by heavy goods vehicle (HGV), from overseas port to UK port by sea, and from UK port to UK distributor and hospital by HGV. The emission factor for hospital waste incineration was based on work by Rizan *et al*.^6^ In the case of manufacture, the CO_2_e emissions associated with the manufacture of each item’s materials (e.g., stainless steel, plastics) were included but, due to a lack of data, the emissions related to the transport of the materials from source to factory and manufacturing the actual item (e.g., energy usage for machinery) were excluded.

### Updating the surgical set

A revised appendicectomy set was created that included:

• Additional reusable instruments (all sets): Johann grasper (B. Braun Aesculap) to complement the existing Johann grasper in the set.
• New reusable ports (all sets): Two 5mm ports and one 12mm port with trocars (B. Braun Aesculap) to replace the existing reusable metal ports.
• Reusable replacements for missing instruments from sets (one set each): one pair of laparoscopic scissors (B. Braun Aesculap) and one pair of Maryland bipolar forceps (B. Braun Aesculap).
• Rationalisation of the set by removing two rarely used graspers.

A trial was conducted with the new reusable ports and written user feedback acquired to confirm that colleagues were satisfied with the reusable port functionality before purchase.

### Reaudit

A reaudit of disposable surgical instrument usage during 22 laparoscopic appendicectomies assessed the financial and environmental impact of the new surgical set compared with the results from the initial audit. The same carbon footprint analysis methodology used above was repeated for the reusable items, which also took into account the predicted number of uses before disposal (manufacturer-stated expected lifespan 500 uses). Sterilisation was not considered in the assessment as the reusable items were added to the existing surgical tray and therefore did not require additional/further sterilisation.

### Statistical analysis

Fisher’s exact test was used to compare the number of appendicectomies that did not require use of one or more of the five aforementioned single-use surgical instruments before and after the set update. The median number of single-use surgical instruments used per case pre- and post- set-update was compared using Mann–Whitney *U* test.

## Results

### Staff surveys

The first survey had 21 responders (50% response rate) comprising seven consultants, 12 registrars and two senior house officers (SHOs). Over half of surgeons (57%) rarely or never used the existing reusable ports on laparoscopic surgical sets due to concerns over ports slipping during use (74% of responders), the trocar being too sharp for safe insertion (47% of responders), laparoscopic instruments getting caught in the ports (37% of responders) and air leaks around the ports impacting the pneumoperitoneum (32% of responders). Colleagues self-reported regularly opening disposable Johann graspers and laparoscopic scissors (85% and 75%, respectively). The laparoscopic appendicectomy set was voted as the set most in need of updating.

A second survey on the existing laparoscopic appendicectomy set was distributed to the 23 registrars and SHOs as the primary users of the appendicectomy set (14 responses, 61% response rate). Only 50% of responders were satisfied with the current set. Colleagues self-reported regularly opening disposable Johann graspers and ports (69% and 61% of responders, respectively). Of note, a disposable Johann grasper was routinely opened during cases as most surgeons reported performing laparoscopic appendicectomy using two Johann graspers, but only one reusable Johann was on the existing appendicectomy set.

### Audit

The audit found that 109 single-use instruments were used in 25 appendicectomies (Table 1). Every case required the opening of single-use instruments (median four instruments per case, range two to six). Disposable ports and an additional Johann graspers were opened in every case. Scissors and Maryland forceps were opened occasionally, likely because one set was missing a Maryland and one set had scissors out for repair during the audit window.

**Table 1:** Audit of disposable instrument usage in 25 laparoscopic appendicectomies

Disposable instrument	12mm port	5mm port	Johann	Scissors	Maryland	Total
Number used	26	49	26	7	1	109
Percentage of cases (%)	100	96	100	28	4	100

### Financial and environmental cost of current practice

Focusing on the five most commonly opened disposable instruments (12mm ports, 5mm ports, Johann graspers, laparoscopic scissors and Maryland forceps), and based on hospital sterilisation department data estimating 500 appendicectomies performed per year at the trust, it was estimated that 2,180 single-use instruments were being used annually. These instruments have a financial cost of £49,656 and an environmental cost of 692kg CO_2_e (Table 2, Supplementary material).

**Table 2:** Estimated financial and environmental cost of using disposable instruments in laparoscopic appendicectomies

Disposable instrument	12mm port	5mm port	Johann	Scissors	Maryland	Total
Number of items	520	980	520	140	20	2,180
Cost per item (£)	£22.80	£12.00	£42.00	£24.00	£42.00	–
Total cost per annum (£)	£11,856	£11,760	£21,840	£3,360	£840	£49,656
kg CO_2_e per item	0.325	0.174	0.505	0.475	1.177	–
Total kg CO_2_e per annum	168.9	170.9	262.4	66.5	23.5	692.2

CO_2_e = CO_2_ equivalent

### Updating the surgical set

**Figure 2:**
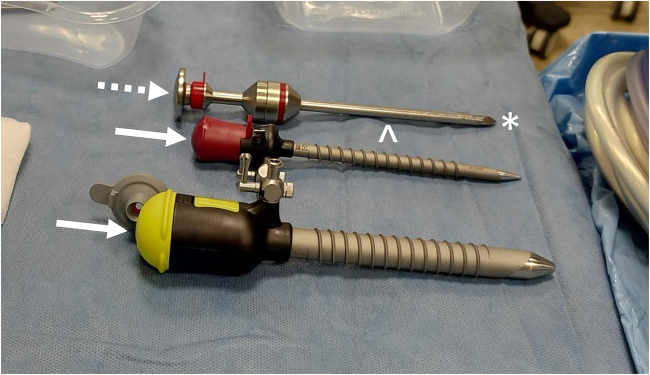
Trial of new reusable laparoscopic ports New reusable ports (line arrows) were purchased to replace the existing reusable ports (dotted arrow) to address concerns regarding the sharpness of the existing trocar (*) and air leak due to lack of threading on the existing ports (^).

A new model of reusable ports was selected that addressed surgeons’ reasons for not using the existing reusable ports (Figure 2). Other instruments were added to the set on request from colleagues and rarely used instruments removed. The final set changes were: new reusable 12mm port with trocar, two new reusable 5mm ports with trocar, additional Johann grasper, additional heavy needle holder, two additional large Langenbeck retractors, removal of two rarely used graspers and removal of old reusable ports. The total investment cost was £19,730.53 and 47.7kg CO_2_e (Supplementary material). The financial and environmental cost of sterilising the set remained unchanged as the size of the set tray remained the same.

### Reaudit

The reaudit found that 31 disposable instruments were used in 22 appendicectomies (range 0–5) (Table 3). There was a significant increase in the number of appendicectomies performed that did not require any of the five disposable instruments (0% versus 41%, *p*=0.0004). The median number of disposable instruments per case also significantly reduced (four versus one, *p*<0.00001). Maryland forceps were used more often in the reaudit, likely because one set had its Maryland out for repair during the audit window.

**Table 3:** Reaudit of disposable instrument usage in 22 laparoscopic appendicectomies after revising the surgical set

Disposable instrument	12mm port	5mm port	Johann	Scissors	Maryland	Total
Number used	7	15	4	2	3	31
Percentage of cases (%)	27	36	18	9	14	59

### Financial and environmental impact of updating the set

There are seven appendicectomy sets in circulation at the trust. Presuming a minimum of 500 uses per reusable instrument and a stable incidence of 500 appendicectomies per year, the new ports are expected to have a seven-year life cycle and be used in 3,500 appendicectomies. Based on practice before updating the set, the cost of disposable instrument usage during laparoscopic appendicectomies was estimated to be £347,592 and 4,845kg CO_2_e over seven years.

The financial and environmental impact of updating the laparoscopic appendicectomy set was estimated by extrapolating the results from the reaudit (Table 4). The revised set is projected to reduce annual disposable instrument usage from 2,180 to 705 instruments (68% reduction), saving over £219,000 and three tonnes of CO_2_e over seven years (annual savings of £31,350.32 and 0.43 tonnes of CO_2_e). Per appendicectomy, this reduces the additional financial cost from £99.31 to £36.61 (63% reduction) and environmental cost from 1.38 to 0.52kg CO_2_e (62% reduction) per case. Based on the projected annual savings, the capital investment for new reusable ports and additional instruments (£19,730) will pay for itself after eight months.

**Table 4:** Projected financial and environmental impact of revising the appendicectomy set

Reusable instruments	Quantity	Uses	Financial cost (£)	Environmental cost (kg Co_2_e)
Reusable 12mm port + trocar	7	2,386	5,023.27	6.6
Reusable 5mm port + trocar	14	1,114	5,135.75	10.1
Reusable Johann grasper	7	2,864	5,895.89	18.0
Reusable laparoscopic scissors	1	3,182	998.23	2.5
Reusable Maryland forceps	1	3,023	1,563.69	2.5
Mayo needle holder	7	2,625	400.68	2.4
Large Langenbeck retractors	14	2,625	713.02	5.6
Single-use instruments	Quantity	Uses	Financial cost (£)	Environmental cost (kg Co_2_e)
Disposable 12 mm port + trocar	1,114	1	25,399.20	362.1
Disposable 5mm port + trocar	2,386	1	28,632.00	415.2
Disposable Johann grasper	636	1	26,712.00	321.2
Disposable laparoscopic scissors	318	1	7,632.00	116.4
Disposable Maryland forceps	477	1	20,034.00	561.4
Projected annual and life cycle costs			Financial cost (£)	Environmental cost (kg Co_2_e)
Projected cost over seven years (previous appendicectomy set)			347,592.00	4,845.0
Projected cost over seven years (revised appendicectomy set)			128,139.73	1,824.0
Projected savings over seven years			219,452.27	3,021.0
Projected annual average saving			31,350.32	431.6

CO_2_e = CO_2_ equivalent

## Discussion

This susQI project demonstrated that updating a laparoscopic appendicectomy set can significantly reduce disposable instrument reliance. This translates to marked projected financial savings and reduced carbon emissions. Around 45,000 appendicectomies are performed annually in England.^7^ Presuming that 95% of these are performed laparoscopically,^8^ if these results were reproduced nationally this would result in an annual financial saving of £2.68 million and the avoidance of 36.9 tonnes of CO_2_e.

A recent systematic review identified single-use consumables as a significant contributor to the carbon footprint of surgical procedures.^9^ Recent studies have investigated the environmental and financial impact of updating specific surgical sets to reduce consumable usage. Boag and colleagues performed an extensive streamlining of their appendicectomy set and process, including switching to reusable gowns and drapes and using a gasless laparoscopy device.^10^ Replacing single-use instruments with reusable instruments had a forecast annual saving of £9,567 and 0.74 tonnes of CO_2_e based on a predicted 80% reduction in disposable instrument use. Rizan and Bhutta performed a life cycle assessment of hybrid (part reusable) laparoscopic ports, scissors and clip applicators during laparoscopic cholecystectomies and found that hybrid instruments reduced per-operation CO_2_e emissions from these instruments by 76% (7.19 versus 1.75kg CO_2_e) with a 53% reduction in financial cost (£282 versus £131).^11^ Kodomuri and colleagues streamlined a carpal tunnel surgery kit so that a smaller surgical set tray could be used. Their revised set resulted in a 14.7kg CO_2_e reduction and saved £33.71 per case (35% cost reduction).^12^ All of the above studies updated existing surgical sets rather than separately sterilising and packaging reusable instruments, likely because this is associated with reduced carbon emissions and financial costs from sterilisation compared with streamlining individual instruments.^13^ Our results and the above studies show that optimisation of surgical sets leads to financial savings even when factoring in the capital investment required for new equipment.

During our project, barriers encountered were initial belief that the appendicectomy surgical set had been updated recently, hesitance from colleagues on the impact of updating the set, and concerns regarding securing capital to purchase new instruments. Demonstrating the financial and environmental cost of current practice using audit provided data to present to the trust board and finance department, facilitating the acceptance of our business case. Involving colleagues in all decision-making regarding equipment purchase and set changes ensured buy-in from colleagues and positive feedback following the trial of the new ports.

This study has several limitations. As the number of appendicectomies audited was relatively low (25 and 22, respectively), there is a significant degree of uncertainty in the actual projected savings over a seven-year life cycle. The financial and environmental costs from Maryland forceps in the revised appendicectomy set is likely to be overestimated as one set was missing its Maryland during the reaudit window. The estimated financial saving does not include the money saved from avoiding the incineration of an estimated 220kg of disposable instruments annually. Lastly, this project has calculated a CO_2_e reduction from replacing some consumables frequently used in laparoscopic appendicectomies, rather than calculating the entire carbon footprint of a laparoscopic appendicectomy. A full carbon costing would need to account for many sources of emissions, such as theatre energy usage, surgical equipment, anaesthetic gases, medications, etc (Figure 1).

In conclusion, this project has demonstrated that revising a laparoscopic appendicectomy set to include new reusable ports and additional reusable instruments leads to a significant reduction in the use of disposable surgical instruments, resulting in marked projected financial and CO_2_e savings. The authors recommend regular review of surgical sets to optimise the included equipment and reduce reliance on single-use surgical instruments.
